# Effects of an active exoskeleton on the muscle activity of the erector spinae and biceps femoris muscles during lifting with symmetric stoop and squat technique

**DOI:** 10.3389/fbioe.2026.1631785

**Published:** 2026-05-12

**Authors:** Jens Hasenmaier, Tobias Siebert, Dominik Mayer, Norman Stutzig

**Affiliations:** 1 Motion and Exercise Science, University of Stuttgart, Stuttgart, Germany; 2 Stuttgart Center for Simulation Science, University of Stuttgart, Stuttgart, Germany

**Keywords:** active, apogee, electromyography, exoskeleton, lower back, muscle activity, musculoskeletal disorders

## Abstract

**Introduction:**

Active exoskeletons are increasingly used to reduce muscle activity and support lifting tasks. However, the effectiveness of exoskeletons can vary depending on the lifting technique. This study investigates the effects of a commercially available active exoskeleton (Apogee) on muscle activity during two different lifting techniques: stoop and squat.

**Methods:**

17 healthy young adults (8 male, 9 female) participated in the study. Muscle activity was measured in the M. erector spinae (MES) and M. biceps femoris (MBF) using EMG while lifting a 15 kg box in four conditions: 1) without exoskeleton, 2) exoskeleton in passive mode, 3) 50% support and 20% counterforce, 4) 100% support and 60% counterforce.

**Results:**

The MBF activity was 29% lower during the squat technique compared to the stoop technique (F_1_,_16_ = 20.53, p < 0.01, ղ_P_^2^ = 0.56). MES activity was similar across both techniques (F_1,15_ = 0.53, p = 0.48, ղ_P_^2^ = 0.03). When pooling data across techniques, the exoskeleton significantly reduced MES (F_1,45_ = 18, p < 0.01, ղ_P_^2^ = 0.53) and MBF activity (F_1,48_ = 10.2, p < 0.01, ղ_P_^2^ = 0.39), with higher support levels leading to greater reductions in muscle activity. The stoop technique benefitted more from the exoskeleton, showing significant reductions in MES activity (10%–27% MVC) and MBF activity (7%–10% MVC). In contrast, squat lifting showed a significant reduction in MES (10%–17% MVC) activity but no significant reduction in MBF (2%–3% MVC) activity.

**Discussion:**

The results indicate that the exoskeleton provides greater benefits during the stoop technique, where the lifting motion primarily involves hip extension. The reduction in muscle activity supports the potential of exoskeletons in reducing muscle load and preventing work-related musculoskeletal disorders (MSDs). However, the squat technique showed limited improvements, suggesting that exoskeletons may need to be adapted for different lifting tasks. Future exoskeletons should aim to dynamically adjust their support based on the specific lifting technique to maximize effectiveness.

## 1 Introduction

Musculoskeletal disorders (MSD) are one of the most common work related diseases and have a multifactorial aetiology (Schneider and Irastorza, 2010). MSD describe injuries and dysfunctions that can affect the muscles, bones, nerves, tendons, ligaments, cartilage and discs (Da Costa and Vieira, 2010). In the context of working environment, they are also referred to as work related MSD. Causal relationships between physical stress and work related MSD can be observed (Bernard and Putz-Anderson, 1997). Especially MSD in the lower back area have a high prevalence and are the most common worldwide (Holzgreve et al., 2023). As a result, 27% of absences from work are attributed to MSD. In addition, MSD-related diseases lead to longer periods of incapacity to work. Consequently, 30%–40% of the absences from work could be avoided through prevention (Thiehoff, 2002). One possible cause of MSD in the lower back is repeated lifting of weight in different postures (Da Costa and Vieira, 2010).

**FIGURE 1:**
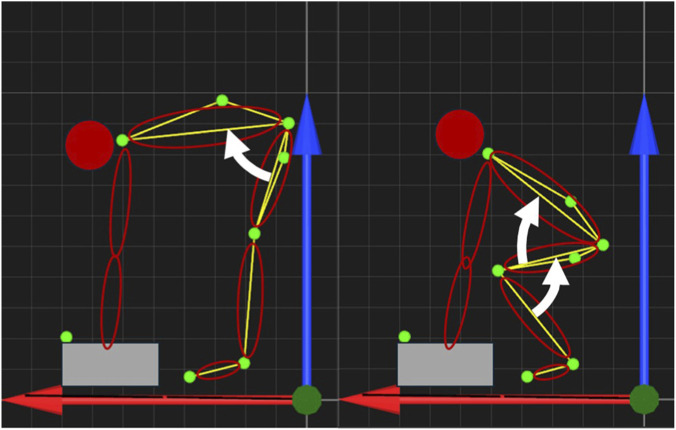
Schematic representation of the stoop- (left) and the squat-technique (right) based in the QTM data. The main movement is indicated by white arrows. The green dots correspond to the positions of the optical markers. Red and blue arrows indicate the horizontal and vertical directions, respectively.

There exist mainly two different techniques to lift weights: the squat-technique and the stoop-technique. While the squat-technique describes a lifting primarily through bending the knees and keeping the trunk upright, the stoop-technique is characterized by slightly bended or extended knees and a forward bend of the trunk (Figure 1). Therefore, the squat-technique can be quantified as mainly (∼135°) knee flexion and minor (<30°) trunk flexion, while the stoop-technique can be quantified as minor (<45°) knee flexion and mainly (∼90°) trunk flexion. The contribution of the hip and knee muscle activation patterns involved in each lift therefore differs significantly (Washmuth et al., 2022). Furthermore, studies have shown that the difference in back loading between the techniques are only slight. Aasa et al. (2022) described that although the trunk leans further forward with the stoop-technique, the orientation of the individual vertebral bodies shows only minor differences. Garg and Herrin (1979) observed only small differences in the load on the intervertebral discs. However, van der Have et al. (2019) found higher peak moments in the L5S1 joint and in the hip joint as well as higher activity of the back muscles (erector spinae iliocostalis and longissimus) when lifting weights using the stoop technique. One aim of MSD prevention could be to minimise the muscle activity of the back and hip extensors and to reduce the stress on joints and internal tissues, such as cartilage and intervertebral discs when lifting heavy loads. Although, the evidence does not favour either technique, in practice many therapists generally prefer the squat technique. Accordingly, the squat technique is the most recommended technique by therapists (Nolan et al., 2018; Smith and Thomson, 2020). Exoskeletons are an interesting and appropriate approach to reducing muscle activity when lifting heavy loads (Govaerts et al., 2021). Exoskeletons are designed to decrease the stress on the users body as reaction on external load and prevent incapacity for work (Schick, 2018). A distinction can be made between active exoskeletons, which are supported by an electric motor, for example, and passive exoskeletons, which are supported by springs or cables (Kaupe et al., 2021). A further distinction could be made into powered, unpowered and quasi-passive exoskeletons. Powered exoskeletons can be described as devices with components that can generate torque. Unpowered exoskeletons, on the other hand, use passive elastic structures to store and release energy. Quasi-passive devices also use passive elastic structures to store and release energy, but with lower power energy requirements than powered/active exoskeletons (Qu et al., 2025).

In a review, Bär et al. (2021) concluded that exoskeletons can relief acute physical stress of the user. 20 of the reviewed studies especially investigated back supporting exoskeletons (BSE) showed a reduction of muscle activity of the back and hip extensor muscles like the m. Erector spinae (MES) and m. Biceps femoris (MBF). Recent studies support this summary statement by reporting a reduction in EMG activity of MES by 18%–27% (Glitsch et al., 2020) and 12%–15% Huysamen et al. (2018) as well as a reduction in EMG activity of MBF by 5% Huysamen et al. (2018) while lifting with a BSE. Studies are increasingly investigating the effect of actively powered BSE (e.g., with an electric motor) (Johns et al., 2024; Walter et al., 2023; Mayer et al., 2025). In particular, Walter et al. (2023) observed a reduction in the muscle activity of the MES with each support level of a “Cray X” (German Bionic Systems GmbH) device when lifting using the stoop technique. Mayer et al. (2025) also observed a reduced MES and MBF activity, especially while using the highest support level of the exoskeleton “Apogee”. Most studies were performed using the stoop technique (Walter et al., 2023; Mayer et al., 2025) or did not specify the lifting technique (Huysamen et al., 2018; Glitsch et al., 2020; Johns et al., 2024). However, to our best knowledge, no study has yet been conducted to compare the two techniques (stoop vs. squat) using active exoskeletons regarding the neuromuscular activity of MBF and MES.

While the MBF supports the hip extension in both techniques, the MES is a central agonist, especially during the stoop technique, which leads to an activation of the MBF and MES during lifting (Washmuth et al., 2022). Although exoskeletons are known to assist with lifting and reduce neuromuscular activity in key muscles (MES, MBF), it remains unclear whether they provide greater benefit during stoop lifting or squat lifting. This information is needed to assess and improve movement support by active exoskeletons to prevent work-related MSD.

Therefore, this study aims to analyse and compare the neuromuscular activity of the MES and MBF during two lifting techniques (stoop vs. squat), both with and without an active exoskeleton. To clearly distinguish the movement and the contributions of the relevant muscles (MES, MBF), a 3D video analysis is carried out in combination with a recording of muscle activity using EMG. It was hypothesised that the exoskeleton reduces EMG activity of back- and hip extensor muscles equally for both techniques. Further the contribution of this study is:

- To investigate and compare the effects of the exoskeleton on the muscle activity of different muscles while symmetric lifting with the stoop- and squat-technique
- To improve the understanding of how the exoskeleton influences the different lifting techniques and to find out whether one of the techniques could benefit more from the use of the exoskeleton.

## 2 Methods

### 2.1 Participants

19 young and healthy adults (sex: nine male and ten female; age: 21.5 ± 2.5 years; height: 174.4 ± 8.1 cm; weight: 68.6 ± 9.8 kg) participated in the study. None of them had experience with exoskeletons. Two subjects had to be excluded due to technical issues (unstable EMG-signal because of faulty electrodes), therefore 17 subjects were included in the evaluation. The subjects were informed about possible risks of the experiment and gave their written consent. The study was approved by the University of Stuttgart Ethics Committee (AZ. 24–034) and was conducted in accordance with the latest declaration of Helsinki.

### 2.2 Exoskeleton

**FIGURE 2:**
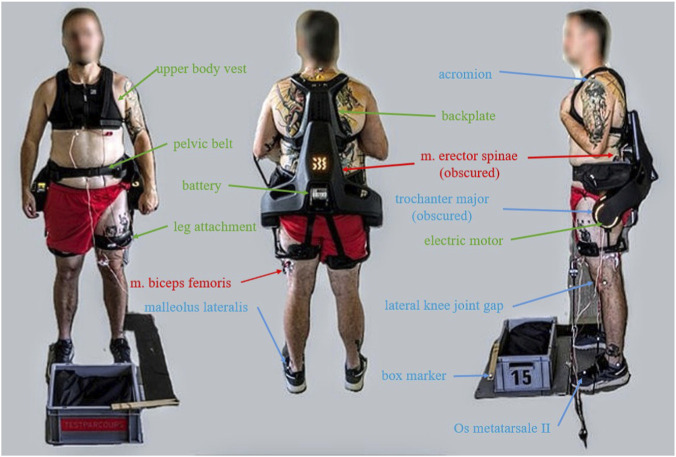
Subject wearing the Apogee exoskeleton. The 15 kg box (40 × 30 × 22 cm) to be lifted can be seen in the left and right images. EMG electrodes (red), optical markers (blue) and parts of the exoskeleton (green) are marked. A detailed illustration of the Apogee can be found in the Supplementary Material.

In this study, the commercially available active exoskeleton Apogee (German Bionic Systems) was used (Figure 2). This exoskeleton is a successor of the Cray-X system used in Walter et al. (2023) and enables a better support due to a stronger electric motor and tighter upper body attachment. The Apogee weights about 8 kg and is worn like a hiking backpack. The weight of the device is mainly supported by a lap belt so that it rests mainly on the iliac crest. In addition, the exoskeleton is fixed to the user with a chest waistcoat and two leg attachments. The position and inclination of the trunk are measured by the device with inertial measurement units. This allows the device to recognise the user’s movement so that the support in form of torque can be applied to the hips at the right time. The exoskeleton offers two types of support (extension support and counterforce). The counterforce, that is applied when bending down to support the user while lowering the weight, and the actual extension support that is applied when straightening up again. Each type of support can be set from 0% to 100% in increments of 10%.

### 2.3 Procedure

Before the experiment started, a measurement to capture the EMG signal of the maximum voluntary contraction (MVC) of the MES and MBF was conducted. To measure the MVC of the MES the subject had to lie flat on their stomach and try to straighten their back against a fix resistance for 3 seconds. This was done two times with a 60 s break in between. The MVC of the MBF was measured in the same position, but with a knee angle of 90°. Then the subjects had to flex their knee against a fix resistance (Konrad, 2011). This was also done two time with a 60 s break in between. The measurement with the higher EMG value was set as the MVC value and taken into account in the further evaluation. When performing the maximum voluntary contractions, we followed the recommendations of Gandevia (2001).

**FIGURE 3:**
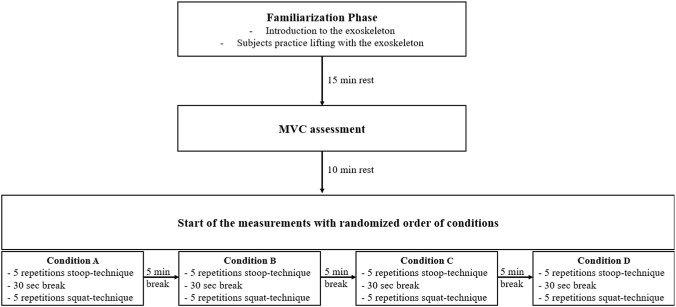
Schematic structure of the study procedure.

To evaluate the impact of the exoskeleton and different levels of support on the two lifting techniques, the muscle activity of the MES and MBF was measured using EMG. Therefore, the subjects were asked to lift a 15 kg box (40 × 30 × 22 cm) five times with each technique in the four conditions: 1) without exoskeleton, 2) with exoskeleton in passive mode (no active support), 3) with active exoskeleton (50% support and 20% counterforce), and 4) with active exoskeleton (100% support and 60% counterforce) from the ground to standing upright. The choice of balance between level of support and counterforce was made based on a previous study with an earlier model of the exoskeleton (Walter et al., 2023). The choice of the weight and repetitions was oriented in line with Huysamen et al. (2018) and Walter et al. (2023). Therefore, five repetitions were selected to ensure that enough repetitions were available for evaluation and that the test subjects did not experience fatigue. The order of the conditions was randomised but the subjects either started with the condition without exoskeleton or ended the experiment without exoskeleton. This was done to ensure that there was no complication with the cable driven EMG device while putting the exoskeleton on or taking it off during the experiment. The conditions always started with five repetitions stoop-technique and, after a 30 s break, ended with five repetitions squat-technique. Between the conditions there was a 5-min break to recover. The lifting speed was given by a metronome which truck at 45 bpm. Each part of the movement (e.g., becoming upright from the ground) had to be done in one beat. To ensure correct handling with the exoskeleton and a correct execution of the technique, the subjects were introduced to the exoskeleton and had a 15-min familiarization phase with it before the experiment started. After the familiarization phase, a break of 15 min was taken to avoid fatigue effects. The schematic structure of the study procedure can be seen in Figure 3.

### 2.4 Data recording

For measuring the muscle activity, bipolar surface EMG measurements were conducted on the left MES (m. longissimus) and left MBF during lifting. Therefore, the skin over the muscle was prepared by shaving, abrading with sandpaper and cleaning with alcohol to increase the skin conductivity and the signal quality (Hermens, 1999). Then, the muscle were located according to the SENIAM recommendations (Hermens, 1999). Consequently, two self-adhesive EMG electrodes were placed with a 2 cm interelectrode distance on the MES about two fingers wide outside the spinous process of the first lumbar vertebra. To record the MBF signal, two electrodes were placed in the centre between the ischial tuberosity and the lateral epicondyle of the tibia. In addition, a reference electrode was placed on the medial malleolus. Generally, there was enough space to place the electrodes despite the attachment points of the Apogee, but in some cases the placement had to be shifted a little bit, to prevent pressure on the electrodes by the attachments. In these cases guidance for electrode positioning by Barbero et al. (2012) was followed. The EMG signal was measured with the wired measuring device MP160 from Biopac Systems (Goleta, USA). The EMG data was recorded with 2 kHz, filtered using a bandpass filter (10 Hz and 500 Hz) and stored on a computer (Stutzig and Siebert, 2016).

The movement of the subjects was tracked using an infrared camera system with four cameras (Qualisys, Gothenburg, Sweden). Reflective markers were placed on the left side of the body at anatomical landmarks as follows: Os metatarsale II, malleolus lateralis, lateral knee joint gap, trochanter major, on a line between trochanter major and knee joint gap, acromion and one on a line between trochanter major and acromion, plus an extra marker on the box to determine the start of the lifting phase (Figure 1). The tracking was done with the software Qualisys Track Manager (Qualisys, Gothenburg, Sweden) and recorded with 340 Hz. The movement tracking and EMG recording was synchronized via a trigger that sent a signal to a separate analog channel of the Biopac system.

### 2.5 Data processing

For both techniques during each condition only the last four of the five repetitions were evaluated. The first lift of each five repetitions served as a preparation trial for the following lifts, since the subjects did not know the exact level of support for the condition. The time period in which the box was lifted was analysed, only. Therefore, the analysed movement was defined from the beginning when the box went up to the highest point of the box.

The EMG data and the kinematic data were processed using Matlab® 2024a (MathWorks, Massachusetts, United States). The EMG data of the experiment and of the MVC measurements was fully rectified and smoothed using a moving average over 150 m. In addition, the signal was normalized to the peak value of the MVC measurement. Afterwards the maximum EMG value during the lifting period was determined for each subject and lift.

The kinematic data was used to define the starting and final position and as a control of the techniques. Since there were only four cameras available and the exoskeleton repeatedly occluded markers during the movement, only one side of the body was captured and the 3D coordinates of the markers were reduced to the sagittal plane, which allowed a 2D vision on the relevant angles (Figure 1).

### 2.6 Statistical analysis

The data are presented as mean value and standard error (SE). For further analysis, the data were tested for a normal distribution using the Kolmogorov-Smirnov test. All data were normally distributed. A 2 (TECHNIQUE [stoop vs. squat]) X 4 (SUPPORT [without Exo vs. with Exo (0/0%) vs. with Exo (50/20%) vs. with Exo (100/60%)]) repeated-measures analysis of variance (rANOVA) was conducted for the EMG data. Effect size was determined using partial eta squared (ղ_P_^2^).

${ղ}_{P}^{2}={\text{SS}}_{\text{between}}/\left({\text{SS}}_{\text{between}}+{\text{SS}}_{\text{error}}\right),$ where.

${\text{SS}}_{\text{between}}$ –sum of squares of the interested effect.

${\text{SS}}_{\text{error}}$ –sums of squares of the error of the interested effect.

The effect sizes were classified as low (ղ_P_^2^ = 0.01), medium (ղ_P_^2^ = 0.06) and large (ղ_P_^2^ = 0.14) (Cohen, 1988). When the rANOVA demonstrated significant main effects or interactions, *post hoc* analyses were performed using the Tukey honestly significant difference test. The significance level was set at p < 0.05. All analyses were performed using STATISTICA (Version 14.1.0.8, StatSoft GmbH, Hamburg, Germany).

## 3 Results

The MBF data of all subjects (n = 17) could be considered for the study. However, the MES data had to be excluded for one subject due to artefacts (n = 16).

### 3.1 Comparison of pooled data: technique

**FIGURE 4:**
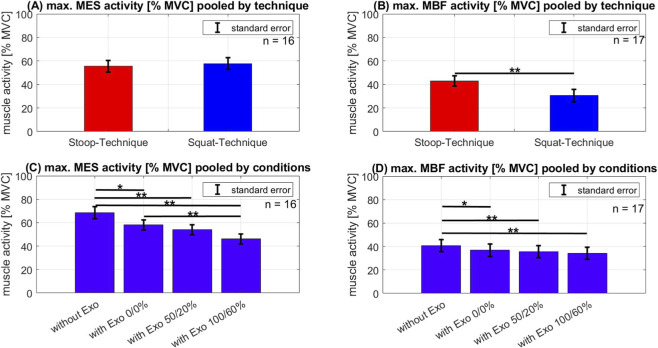
Mean and standard error of muscle activity **(A,C)** of the M. erector spinae (MES) and **(B,D)** of the M. biceps femoris (MBF) when lifting a load. The EMG data were pooled by technique **(A,B)** and pooled by conditions **(C,D)**. Significance: *p < 0.05; **p < 0.01.

In order to investigate the general differences in muscle activity between the two lifting techniques, all data (four conditions) were pooled for each lifting technique. The comparison of the lifting techniques showed a 29% lower MBF activity in the squat technique compared to the stoop technique (Figure 4B, F_1,16_ = 20.53, p < 0.01, ղ_P_^2^ = 0.56). In contrast, MES activity of both lifting techniques is similar (Figure 4A, F_1,15_ = 0.53, p = 0.48, ղ_P_^2^ = 0.03).

### 3.2 Comparison of pooled data: level of support

In order to make a general statement about the influence of the exoskeleton independent of the lifting technique, the data from the stoop and squat techniques were pooled. In this regard, a significant effect on MES activity was observed (F_3,45_ = 17.00, p < 0.01, ղ_P_^2^ = 0.53). A comparison of the support conditions of both pooled techniques revealed a significant difference for MES activity between the first condition without exoskeleton and all others (Figure 4C). More precisely, compared to the situation without an exoskeleton, muscle activity was reduced by 15% MVC (p_1-2_ = 0.01) when the passive exoskeleton was applied, by 21% MVC (p_1-3_ < 0.01) when the 50% support was provided, and by 33% MVC (p_1-4_ < 0.01) when the 100% support was provided.

Regarding the MBF activity, a significant effect was also observed (F_3,48_ = 10.20, p < 0.01, ղ_P_^2^ = 0.39). The *post hoc* analyse of the MBF activity demonstrated a significant difference between the first condition without exoskeleton and all the others (p_1-2_ = 0.02, p_1-3_ < 0.01, p_1-4_ < 0.01) (Figure 4D). No significant difference could be measured between the different level of support (passive mode, 50/20% and 100/60%) of the exoskeleton.

### 3.3 Interaction effects: technique and level of support

**FIGURE 5:**
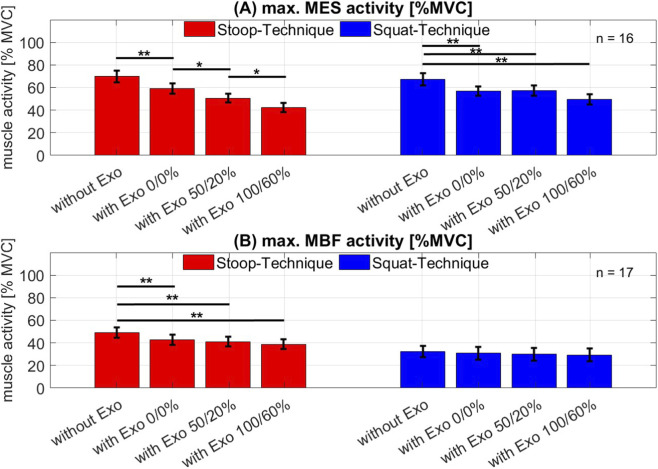
Mean and standard error of muscle activity **(A)** of the M. erector spinae (MES) and **(B)** of the M-biceps femoris (MBF) when lifting a load using the stoop technique (red bars) and the squat technique (blue bars). The single bars demonstrate the 4 conditions: without exoskeleton (without Exo); using exoskeleton with 0% support and 0% counterforce (with Exo 0/0%); using exoskeleton with 50% support and 20% counterforce (with Exo 100/60%); using exoskeleton with 100% support and 60% counterforce (with Exo 50/20%) Significance: *p < 0.05; **p < 0.01.

Analysing the interaction effects between technique and the level of support revealed significant effects for MES (F_3,45_ = 4.47, p < 0.01, ղ_P_^2^ = 0.23) and MBF activity (F_3,48_ = 4.85, p < 0.01, ղ_P_^2^ = 0.23). The comparison of the conditions within the stoop-technique demonstrated a significantly reduced MES activity when using the exoskeleton and with each level of support (Figure 5A, left). While in condition 1) the mean MES activity was 69.8% MVC, it was at 59.2% MVC in condition 2) with exoskeleton, but 0/0% support (p_1-2_ < 0.01). In condition 3) with 50/20% support, it was reduced to 50.7% MVC (p_2-3_ = 0.03) and in condition 4) with exoskeleton and 100/60% support, it was 42.4% MVC (p_3-4_ = 0.04). The MBF activity was only significantly reduced comparing condition 1) without exoskeleton to all the other conditions with exoskeleton (p_1-2_ < 0.01, p_1-3_ < 0.01, p_1-4_ < 0.01) (Figure 5B, left). There was so significant difference between the different level of support.

Using the squat-technique, there was a significant difference in MES activity only in comparison of condition 1) to all the other conditions as well (p_1-2_ < 0.01, p_1-3_ < 0.01, p_1-4_ < 0.01) (Figure 5A, right). Starting in condition 1) without exoskeleton with a mean activity of 67.4% MVC to 49.7% MVC in condition 4). There was no difference between condition 2) and condition 3). But the comparison from condition 4) to condition 2) and 3) showed a tendency for significance (p_2-4_ = 0.99, p_3-4_ = 0.07). The evaluation of the MBF activity during the squat-technique did not show any difference between the conditions.

A summary of the main findings can be found in the Supplementary Table S1.

## 4 Discussion

In this study, we investigated the effect of an active exoskeleton on two different lifting techniques (stoop vs. squat). To our knowledge, this is the first time that such a direct comparison of these lifting techniques has been carried out in combination with an analysis of the effects of the exoskeleton on different muscles during a symmetrical lift. It was shown that the exoskeleton generally has a supportive effect, regardless of the lifting technique. However, due to the different joint kinematics between the lifting techniques, differences were found in the reduction of muscle activity of relevant muscles (MES, MBF). Overall, an increase in the level of support led to a reduction in muscle activity.

### 4.1 General statement of the ratio between support and counterforce

The (extension) support of the Exoskeleton (Apogee) is applied when the users straighten up their back when lifting the weight. While the counterforce supports the user during controlled lowering of the weight. This results in a certain dependency between load and the settings for support and counterbalance. In general, it can be said that the higher the extension support (and therefore lower muscular moments are required for lifting), the higher the counterforce (controlled lowering of the weight). This results in certain settings and recommendations that are practical and based on German Bionic Systems GmbH (Augsburg, Germany) prior experience.

For our two support levels, these are:

- 100% extension support/60% counterforce
- 50% extension support/20% counterforce

Based on a fixed ratio of 100%/60%, one would expect a ratio of 50%/30% at a lower support level. However, we have used the recommended ratio of 50%/20% support. This results in lower support when lowering the load. Since this study focuses exclusively on the lifting motion (and not the lowering motion), the choice of counterforce should not affect the results for the lifting motion.

### 4.2 Classification of the results

The reductions in MES and MBF muscle activity observed in this study (Figures 4, 5) using an exoskeleton are within the range of the reported reductions in MES and MBF activity in the literature. Kermavnar et al. (2021) described an average reduction of MES of 25% MVC and MBF of 5% in their review, and Walter et al. (2023) showed a reduction of the MES activity of about 22% during stoop-lifting with an active exoskeleton. This study was able to measure a reduction of MES activity of about 10%–27% MVC and a MBF activity reduction of about 10% MVC across both lifting techniques. While there was no comparison between lifting technique, Poliero et al. (2022) also showed a 41% MVC decrease in lumbar extensor muscles activity during a dynamic task when using an active exoskeleton and a 16% MVC decrease when using a passive device.

#### 4.2.1 Muscle activity during stoop-technique

A separate evaluation of the techniques showed that wearing the exoskeleton while using the stoop-technique reduced the MES activity by 8%–10% MVC (Figure 5A, left) with each level of support chosen in this investigation. Similar effects were observed by Walter et al. (2023), where muscle activity decreased with higher support level. The activity of the MBF only reduced when wearing the passive Apogee (Figure 5B, left), but no extra reduction could be observed when using higher level of support. In contrast, Frost et al. (2009) observed an increased reduction in MBF activity with increased support of the exoskeleton. But it should be noted that the used exoskeleton in their study was a passive BSE which positioned the leg attachment below the knee joint. Therefore, the different design of the device could lead to different results in MBF activity. To our knowledge there is no other study which analysed the MBF activity while using different levels of support of an active BSE.

#### 4.2.2 Muscle activity during squat-technique

Similar results were obtained by analysing the MES activity while using the squat-technique. Although activity was reduced when using the 100%/60% support, there were no significant results between the individual levels (Figure 5A, right). Even wearing the passive exoskeleton already showed a reduction in activity of about 11% MVC. The evaluation of the MBF activity during the squat-technique did not show any significant reduction of the muscle activity (Figure 5B, right). Wei et al. (2020) also observed a reduction in back muscle activity while using a hip active assisted exoskeleton. But no analysis of different levels of support were made in this study.

#### 4.2.3 Influence of the exoskeleton in passive mode

In both techniques, the muscle activity of MES was already reduced just by wearing the exoskeleton in passive mode during lifting, despite the absence of active support (Figure 5A). It can be assumed that the reduction in MES activity arises due to the attachments’ structures of the Apogee. Van Poppel et al. (2000) described that even passive lumbar orthoses can influence the muscle activity. This influence in the form of a reduction of muscle activity is probably caused by the compression and stabilisation of the trunk. Although these effects are not completely understood in literature, van Poppel et al. (2000) also described a change of movement while wearing these orthoses. Since the Apogee is attached to the pelvis via a pelvic belt, which must be tightened around the lumbar region, the same effects could occur as with lumbar orthoses. Therefore, a change in the movement could lead to different muscle activity. Also, the Apogee is attached to the upper body with a hard backplate and a vest, which does not allow as much trunk flexion as without the device. This means that lesser trunk flexion could lead to lesser muscle activity in MES. Similar effects could be observed in MBF during the stoop-technique (Figure 5B, left). Since, depending on the conception of the exoskeleton, the joint angles can change during movement (Schwartz et al., 2023), it is possible that a different upper body posture changes the lever on the MBF, therefore the muscle activity could also change. But since the MBF is more a supporting hip extensor, a higher level of support by the exoskeleton does not necessarily lead to a higher reduction in MBF activity. At this point, it could be relevant to measure the activity of other hip extensors (e.g., m. Gluteus maximus) additionally.

#### 4.2.4 Differences in techniques and level of support

As the muscle activity of the MES decreased with each level of support during the stoop-technique, the activity during the squat-technique only decreased significantly when comparing with and without the exoskeleton, it can be surmised that while analysing the muscle activity the stoop-technique appears to benefit more from the active back supporting exoskeleton. In addition, a reduction in MBF activity was also observed during the stoop-technique, but not during the squat-technique. Since the squat-technique relies mainly on knee extension and the Apogee support the hip extension, these results are to be expected. Similar results could be observed by Frost et al. (2009) when analysing the muscle activity while wearing a passive back supporting exoskeleton. In addition, van der Have et al. (2019) observed higher peak muscle activity and higher peak moments in hip and knee joints during squat lifting without exoskeleton, while the peak moments on L5S1 did not differ between stoop- and squat-technique. Tröster et al. (2022) also found in their analysis with a human body model and a generic BSE model that squat motions without the BSE model induce higher back loading than stoop motions. Tröster et al. (2022) also showed that different levels of support in form of different exoskeleton models (weak passive, strong passive, active and optimal assistant) had a higher impact relief on the back loading for squat motions than for stoop motions. This stands in contrast to our findings on the reduction of muscle activity, since the reduction of the MBF and MES activity with exoskeleton was higher during the stoop-technique than during the squat-technique. This difference in the results could be due to the position of the weight to be lifted. Dreischarf et al. (2016) described that the position of the load in relation to the body is a bigger factor than the technique while lifting weights. Furthermore, as Washmuth et al. (2022) have already described, different lifting techniques without an exoskeleton lead to biomechanical differences, e.g., in the posture of spine and thus to different muscle activities and other parameters. Therefore, the use of an exoskeleton may increase the variety of variables that influence the measured parameters for the stress on the human body. In addition to this complexity of physical parameters the impact of exoskeletons on combined physical and cognitive aspects of industrial work performance remains inadequately understood (Govaerts et al., 2023). Cognitive workload parameters may be, for example, reaction time, accuracy, and subjective measures. In a pioneering study on this integrative research approach (Govaerts et al., 2023) found no significant difference in cognitive work performance, during the execution of a simulated material handling task, between the different types of exoskeletons (passive Paexo Back, active CrayX exoskeletons, and NoExo). Moreover Govaerts et al. (2023) also concluded that the Paexo Back may hinder trunk flexion. These findings intensify the need for further ergonomic evaluations and real-world assessments to enhance user comfort and efficacy as Cardoso et al. (2024) concluded in their review. Nevertheless, such comparisons - as in this study or in other studies (Poliero et al., 2022) - can improve understanding of the effects and benefits of using an exoskeleton.

### 4.3 Limitations

The lack of randomization in the order of techniques used is a limitation of the study, as there is a risk of possible fatigue effects. However, we consider this risk to be negligible, as we estimate that lifting a 15 kg box corresponds to less than 20% of the maximum voluntary contraction force (Baechle and Earle, 2008; Chulvi-Medrano et al., 2010). Based on the literature on fatigue research, it is unlikely that this type of load would lead to fatigue at the metabolic or neuromuscular level (Meyerspeer et al., 2012; Enoka and Duchateau, 2008). It should also be noted that the squat-technique performed in our study differs slightly from the technique described by Washmuth et al. (2022). The definition specifies that the inclination of the trunk should be around 30°, but in this study an average inclination of ∼56° was observed, so this would correspond more to a semi-squat variant. This is because the box was in front of the test subjects, so a forward tilt was necessary to reach it, and it was not possible to stand over the box. Nevertheless, even a semi-squat variant is dominated by a more active knee flexion and extension than an active trunk bending, therefore, the comparison with the stoop technique seems to be valid. Next, it should be mentioned that the position of the box in relation to the body was not observed, but this positioning while holding the box can also influence the stress the lifting causes on the body (Dreischarf et al., 2016). Also, this study did not consider any influence the exoskeleton could have on the balance of the user. Despite other studies did not find a significant effect on perceived balance while using passive exoskeletons (Kim et al., 2020), this could be an interesting parameter to investigate, which could be important for user comfort and safety. It should be noted that this study did not analyse any cognitive parameters which could influence the user´s acceptance of the exoskeleton. Further, no metabolic factors were taken into account, which could provide further information about the physical strain. Finally, we want to state out that there was no baseline condition with an empty box and without an exoskeleton conducted. This could be valuable to isolate absolute effects of the exoskeleton as well as basic differences in muscle activation between the used techniques. Also, we conducted our study using the following extension support/counterforce ratios recommended by German Bionic Systems: 100%/60% and 50%/20%. Therefore, it is not possible to make statements about the influence of other counterforces or ratios. To provide a clearer comparison, future studies should include both a baseline condition (no-load/no-exoskeleton) and an analysis of how the counterforce affects the lifting techniques.

### 4.4 Conclusion

In this study, the commercially available active BSE “Apogee” (German Bionic Systems GmbH, Augsburg) was used to investigate the effect of an active BSE on the muscle activity of the MES and MBF during two different lifting styles for a symmetric lift. The results showed that the user appears to benefit more from the exoskeleton when performing the stoop technique than when performing the squat technique. The result is also to be expected, since with the stoop technique the lifting movement comes mainly from hip extension and the exoskeleton used supports this movement with a motor in this joint. Consequently, we found a significant reduction in MES activity during the stoop-technique for different levels of support, with MVC reduction increasing by 8%–10% with each level of support. In addition, a reduction of the MBF activity was observed during stoop-technique.

The study shows that exoskeletons can help to reduce acute muscle strain in MES and MBF during lifting movements and thus potentially contribute to the reduction of work-related MSDs. Yet, since we only measured acute effects, there is still a need for further research, particularly with regard to the long-term effects in practice. In addition, subjective parameters such as user-perceived exertion and user-perceived stability should be further investigated, as these provide insight into user comfort and acceptance. Furthermore, the impact of the exoskeleton on muscle fatigue during prolonged use of the device should be investigated. However, the study also shows that exoskeletons do not provide the same level of support for different types of lifting movements. Since the aim of the exoskeleton is to support the user as best as possible while lifting, results like these show, that it may be beneficial to develop exoskeletons that are able to recognize the specific movement task and adapt their support strategy in the future or to give the user guidelines on how to lift to get the best support.

## Data Availability

The raw data supporting the conclusions of this article will be made available by the authors, without undue reservation.
