# Supplementary material for: Effects of an active exoskeleton on the muscle activity of the erector spinae and biceps femoris muscles during lifting with symmetric stoop and squat technique
Source: Front Bioeng Biotechnol. 2026 May 12;14:1631785. doi: 10.3389/fbioe.2026.1631785 (PMC13201388; doi:10.3389/fbioe.2026.1631785)
Supplement: Supplementary file 1 [file Supplementaryfile1.docx]

Supplementary Material

# Supplementary Figures and Tables

## Supplementary Figures

**
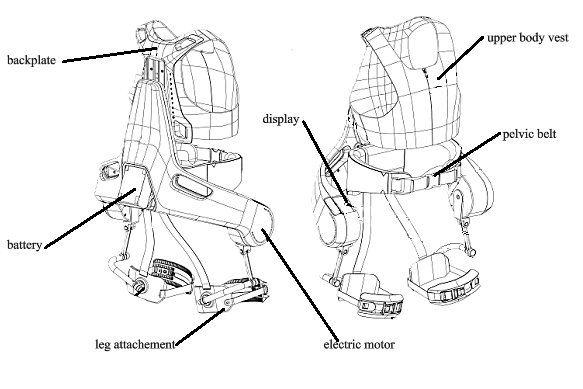
**

**Supplementary Figure S1:** Schematic illustration of the Exoskeleton “Apogee” used in this study

## Supplementary Tables

Table S1: Mean and standard deviation (±) of the maximum muscle activity of the M. erector spinae (MES) and the M. biceps femoris (MBF) in % of maximal voluntary contraction (MVC).

|  | stoop technique | | | | | squat technique | | | | |
| --- | --- | --- | --- | --- | --- | --- | --- | --- | --- | --- |
| support | without | 0/0% | 50/20% | 100/60% | without | | 0/0% | 50/20% | 100/60% |  |
| MES | 69.8 | 59.2 | 50.7 | 42.4 | 67.4 | | 56.9 | 57.3 | 49.7 |  |
|  | (±20.4) | (±18.2) | (±15.4) | (±16.1) | (±21.8) | | (±16.3) | (±18.5) | (±17.8) |  |
| MBF | 49.2 | 42.7 | 41.2 | 38.9 | 32.2 | | 30.8 | 29.9 | 29.3 |  |
|  | (±19.1) | (±18.6) | (±17.4) | (±17.8) | (±20.6) | | (±22.9) | (±22.9) | (±23.5) |  |
